# Gaze Behavior Consistency among Older and Younger Adults When Looking at Emotional Faces

**DOI:** 10.3389/fpsyg.2017.00548

**Published:** 2017-04-13

**Authors:** Laurence Chaby, Isabelle Hupont, Marie Avril, Viviane Luherne-du Boullay, Mohamed Chetouani

**Affiliations:** ^1^Institut de Psychologie, Sorbonne Paris Cité, Université Paris DescartesBoulogne-Billancourt, France; ^2^Sorbonne Universités, Université Pierre et Marie Curie - Paris 06, Institut des Systèmes Intelligents et de Robotique, Centre National de la Recherche Scientifique UMR 7222Paris, France; ^3^Service de Neurochirurgie, Assistance Publique – Hôpitaux de Paris, Groupe Hospitalier Pitié-SalpetrièreParis, France

**Keywords:** aging, emotion identification, facial expressions, visual scanning, gaze patterns, eye-tracking, manifold learning, clustering

## Abstract

The identification of non-verbal emotional signals, and especially of facial expressions, is essential for successful social communication among humans. Previous research has reported an age-related decline in facial emotion identification, and argued for socio-emotional or aging-brain model explanations. However, more perceptual differences in the gaze strategies that accompany facial emotional processing with advancing age have been under-explored yet. In this study, 22 young (22.2 years) and 22 older (70.4 years) adults were instructed to look at basic facial expressions while their gaze movements were recorded by an eye-tracker. Participants were then asked to identify each emotion, and the unbiased hit rate was applied as performance measure. Gaze data were first analyzed using traditional measures of fixations over two preferential regions of the face (upper and lower areas) for each emotion. Then, to better capture core gaze changes with advancing age, spatio-temporal gaze behaviors were deeper examined using data-driven analysis (dimension reduction, clustering). Results first confirmed that older adults performed worse than younger adults at identifying facial expressions, except for “joy” and “disgust,” and this was accompanied by a gaze preference toward the lower-face. Interestingly, this phenomenon was maintained during the whole time course of stimulus presentation. More importantly, trials corresponding to older adults were more tightly clustered, suggesting that the gaze behavior patterns of older adults are more consistent than those of younger adults. This study demonstrates that, confronted to emotional faces, younger and older adults do not prioritize or ignore the same facial areas. Older adults mainly adopted a *focused-gaze* strategy, consisting in focusing only on the lower part of the face throughout the whole stimuli display time. This consistency may constitute a robust and distinctive “social signature” of emotional identification in aging. Younger adults, however, were more dispersed in terms of gaze behavior and used a more *exploratory-gaze* strategy, consisting in repeatedly visiting both facial areas.

## 1. Introduction

Facial emotion processing is widely recognized as a key aspect of everyday life. Accurately decoding emotions in other people's faces is a primary means of non-verbal communication (Ekman and Oster, 1979; Smith et al., 2005), associated with well-being (Carton et al., 1999; English and Carstensen, 2014b) and overall life satisfaction (Ciarrochi et al., 2000). Given its behavioral implications, this ability is also crucial in daily social interactions, being related to more positive social behavior, better social adjustment and adaptation (Izard, 2001; Engelberg and Sjöberg, 2004; Suri and Gross, 2012). Thus, it is not surprising that facial emotion recognition has been extensively investigated across the lifespan (Somerville et al., 2011), with particular focus on aging, which is frequently associated with isolation and social withdrawal. Results from previous studies are generally consistent, showing that older adults are less accurate than younger adults at discriminating and identifying negative facial expressions, especially those of “anger,” “fear,” and “sadness” (Calder et al., 2003; Ruffman et al., 2008; Chaby et al., 2015; Grainger et al., 2015; Templier et al., 2015; Mather, 2016). This emotional shift could emerge stepwise from the fourth decade of life (Calder et al., 2003; Chaby and Narme, 2009). However, the recognition of “joy” and “disgust” does not appear to decline with advanced age, “disgust” being sometimes even better identified by older adults than by younger ones (Calder et al., 2003; Suzuki et al., 2007; Ruffman et al., 2008), even when the faces portrayed low intensity expressions (Orgeta and Phillips, 2007). Given the potential consequences of these difficulties in everyday social interactions, it seems important to understand more about the nature of emotion processing changes in late adulthood.

One dominant explanation for older adults lower performance on negative emotion identification tasks stems from the Socioemotional Selectivity Theory (Carstensen et al., 1999); see for review (Sims et al., 2015). This theory holds that with advancing age, people focus more on emotion regulation goals that can be achieved in the short term (Carstensen et al., 2006; Barber et al., 2016) and on relationships with their closest partners (English and Carstensen, 2014a). Thus, older adults have developed a bias to focus on positive over negative situations or feelings (i.e., known as the “positivity bias”) in order to optimize their affective states (Reed et al., 2014). However, the way in which this “positivity bias” affects the identification of basic facial expressions is still under debate.

Another prominent explanation proposed for age differences in emotion identification, derived from a social neuroscience perspective, is be related to the aging brain model (Cacioppo et al., 2011; Ziaei and Fischer, 2016). Emotion perception involves multiple interconnected brain regions (Lindquist et al., 2012), and some of these brain regions are known to be affected by normal aging (Suzuki et al., 2007). Although a number of studies have investigated the role of various brain regions such as the orbitofrontal cortex, amygdala, insula and temporal regions during the processing of basic emotions, it has been shown that these regions do not work in isolation, but rather form a highly connected network (Pessoa, 2008; Ebner et al., 2012; Lindquist et al., 2012). Several researchers (Calder et al., 2003; Ruffman et al., 2008; St. Jacques et al., 2013) have pointed out that subsequent functional and anatomical changes of these specific brain regions with advancing age may contribute for the difficulty (i.e., for negative emotions such as “anger,” “fear,” and “sadness”) or the ability (i.e., for “joy” or “disgust”) to identify basic emotions. For example, prefrontal cortex atrophy, known as a marker of normal aging, could explain the difficulties encountered by older adults in identifying some facial emotions, in particular faces of “anger” (Baena et al., 2010). Conversely, the preservation of subcortical regions such as the basal ganglia could account for the stability of disgust identification with advancing age (Raz, 2000; Grieve et al., 2005).

**Figure 1:**
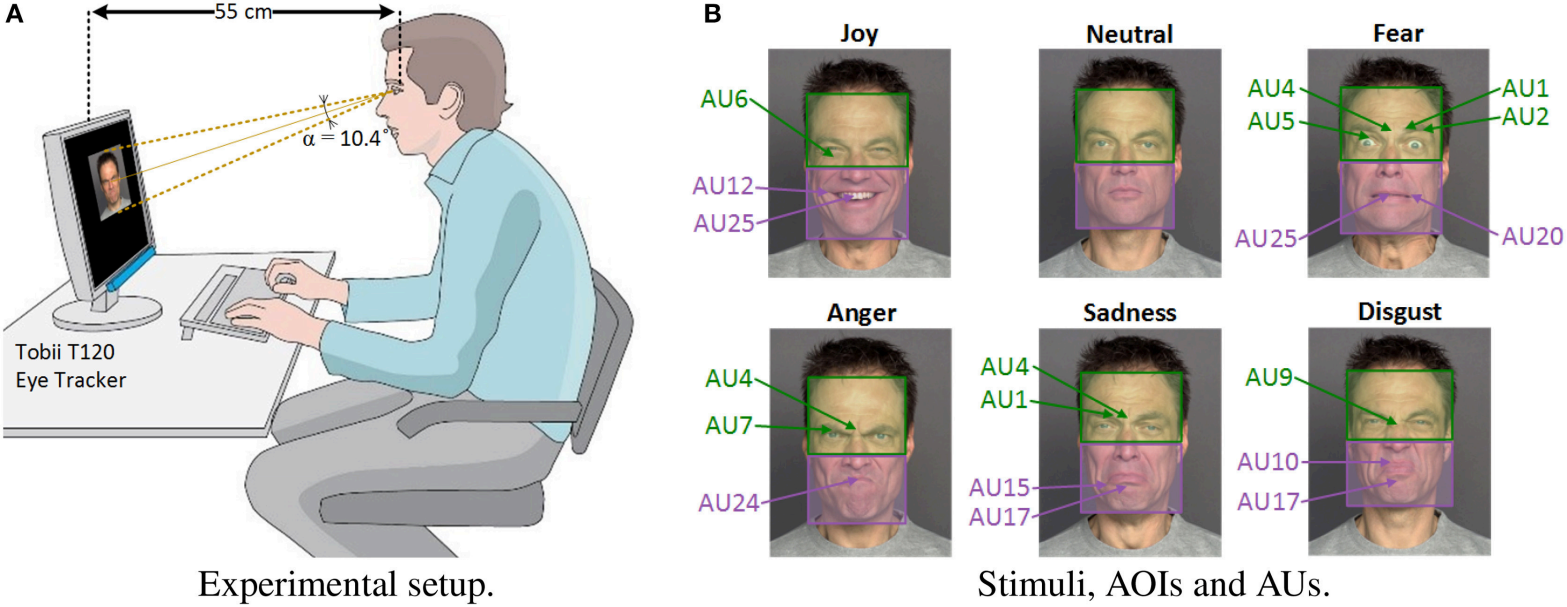
**Experimental setup and stimuli. (A)** Eye-tracking setup used during the experiment. **(B)** Examples of stimuli images. Green and purple colored boxes represent upper-face and lower-face AOIs, respectively. The different Action Units (AU) activated for each facial emotion are shown in green or purple characters, depending on whether they are related to upper-face or lower-face muscles. All facial images are used with permission of the copyright owners (Ebner et al., 2010).

Surprisingly, relatively few studies have explored the possibility that perceptual factors might explain facial emotion recognition difficulties in healthy aging (Orgeta and Phillips, 2007), in spite of many studies indicating age-related changes in visual perception (Bian and Andersen, 2008; Andersen, 2012), especially in face processing (Firestone et al., 2007; Habak et al., 2008; Chaby et al., 2011; Konar et al., 2013). In particular, we showed in a previous study that aging affects some aspects of configural face-encoding processes (e.g., older adults were worse than younger adults at detecting configural changes in the eye region of the face only, but not in the nose–mouth region) which could be related to problems with face recognition (Chaby et al., 2011); see also (Slessor et al., 2013; Meinhardt-Injac et al., 2015). Yet, the processing of such configural information is also relevant to recognize facial expressions (Prkachin, 2003; Calder and Jansen, 2005; Narme et al., 2011; Beaudry et al., 2014) since, when identifying whether a face exhibits a particular emotion, some of its regions may contain more useful and discriminative information than others (Calder et al., 2000; Smith et al., 2005). This is in accordance with the well-known Facial Action Coding System (FACS) by Paul Ekman (Hager et al., 2002), which decomposes facial expressions into small components called Action Units (AUs) anatomically related to the contraction of specific facial muscles. AUs are the building blocks of facial expressions. For example, the facial expression of “joy” usually implies the presence of AU12 “*lip corner puller*,” AU25 “*lips part*” and AU6 “*cheek raise*,” while “fear” involves AU4 “*brow lowerer*,” AU7 “*lid tightener*,” and AU24 “*lip pressor*.” Figure 1B shows the AUs mostly activated in the emotional faces used as stimuli in this paper.

In recent decades, eye-tracking has been used to investigate gaze behavior in childhood and adulthood (Gillespie-Smith et al., 2016). This technique has already shed light on some developmental changes in different attentional processes during facial emotional processing, showing a specific attentional focus on the eye region (Farroni et al., 2002; Leitzke and Pollak, 2016). From a perceptual perspective, it is also an important tool to reveal differences in visual strategies during the lifespan when processing facial information. In young populations, it has been shown that during the exploration of emotional faces gaze mostly focused on a regular triangle-shape over salient parts of the face, namely eyes, nose, and mouth (Eisenbarth and Alpers, 2011).

It seems that the importance of each part of the face depends on the emotion expressed, with more fixations on the lower-face for “joy” or “disgust” and more fixations on the upper-face for “fear,” “anger” or “sadness” (Ponari et al., 2012; Schurgin et al., 2014). However, with advancing age, some studies indicated that the reverse pattern seems to operate. For example, Wong and colleagues reported that contrary to younger adults, older adults looked longer at the lower part of the face when viewing facial expressions (Wong et al., 2005). Furthermore, this over-attention toward the lower-face was found to be correlated with older adults impaired ability to identify certain negative emotions (Wong et al., 2005; Sullivan et al., 2007), especially “fear,” “anger,” and “sadness” whose accurate identification requires examination of the upper part of the face (Calder et al., 2000). However, Murphy and Isaacowitz (2010) reported partially contradictory results, indicating no significant correlation between gaze toward the eyes and age-related emotion recognition deficits. In addition, several studies point out that older adults look globally more at positive and less at negative stimuli than do younger adults (Isaacowitz et al., 2006). Together, these findings suggest that older adults may use a less optimal gaze strategy for extracting key information for facial emotion processing. However, a full explanation for this phenomenon has yet to be established.

The present study was set out to address important but previously under-explored research questions about age-related differences in the strategies used for facial emotion identification by means of non-invasive eye-tracking. Participants' eye movement data were first studied using traditional measures of gaze fixations to determine the overall predisposition of younger and older adults to explore preferentially the upper vs. lower part of the face. Then, finer spatio-temporal dynamics and patterns behind gaze behavior were explored in greater detail. Age-related differences in performance and eye-tracking measures were expected to reveal why older adults perform less well in facial emotion identification for certain emotions and but achieve a comparable performance for some other emotions. More specifically, we address two important research questions: (1) What are the underlying spatio-temporal gaze behavior mechanisms explaining performance differences and similarities obtained by older vs. younger adults in the facial emotion identification task; (2) What are the inter- and intra-group differences and similarities in spatio-temporal gaze exploration strategies while performing a facial identification task.

## 2. Materials and methods

### 2.1. Participants

Initially, 27 younger adults and 36 older adults were recruited for this experiment. However, only 22 of the 27 younger adults (81%) and 22 of the 36 older adults (61%) met the criteria of having a minimal range of 70% of trials with valid gaze information. Loss of eye-tracking data could occur in cases of excessive blinks or sudden changes in body position. In older adults, eye-tracking was more complicated because of eyeglasses, droopy eyelids or watery eyes (Isaacowitz, 2006). Therefore, participants for this study finally included 22 younger adults (20–29 years, 12 females, *M* = 22.18, *SD* = 1.60) and 22 healthy and independently living older adults (60–79 years, 11 females, *M* = 70.4, *SD* = 7.0).

Demographic information for these two groups, including age, years of education and sex ratio, is presented in Table 1. Inclusion criteria required that participants had no history of psychiatric or neurological disorders, which might compromise cognitive functions. All participants were right-handed, according to the Edinburgh Handedness Inventory (Oldfield, 1971) and were required to have: normal hearing; normal or corrected-to-normal vision based on a brief vision screening using the Snellen test (participants whose vision in their best eye was less than 20/30 were excluded); normal score on the Beck Depression Inventory (BDI-II, 21 item version; normal range 0–17; Beck et al., 1996); and performance within the normal range on the Benton Facial Recognition Test^1^ (BFRT, long form; normal range 41–54, Benton and Van Allen, 1968), indicating that basic facial perception skills were intact. Finally, all elderly adults also completed the Mini Mental State Examination test (Folstein et al., 1975), on which they scored above the cut-off score (26/30) for risk of dementia.

**Table 1:** **Participants' demographic characteristics**.

	**Younger adults (*N* = 22)**	**Older adults (*N* = 22)**	***p*-values**
Sex ratio (M/F)	10/12	11/11	–
Age (years)	22.18 ± 1.6	70.41 ± 7.2	<0.001
Education (years)	15.55 ± 0.9	14.77 ± 1.7	0.063
BDI-II (/63)	5.18 ± 3.9	5.27 ± 3.6	0.936
BFRT (/54)	49.86 ± 1.9	49.27 ± 2.0	0.316
MMSE (/30)		29.01 ± 08	

The study was approved by the ethics committee of Paris Descartes University (Conseil d'Evaluation Ethique pour les Recherches en Santé, CERES, n^o^ IRB 2015100001072) and all participants gave informed consent.

### 2.2. Stimuli and apparatus

Stimuli consisted of pictures of facial expressions that were obtained from the FACES database (Ebner et al., 2010). This database was chosen because the stimuli have a high resolution, homogeneous color, and provide good examples of universal emotion categories with a high accuracy of labeling. The faces of 8 models (with an equal number of young and older, male, and female faces) expressing 5 different facial expressions, namely “joy,” “anger,” “fear,” “sadness,” “disgust,” and “neutral”^2^, constituted the set of 48 stimuli used in the experiment.

All stimuli, presented over a black background, were 10 cm high by 8 cm wide and subtended a vertical visual angle α of 10.4° at a viewing distance of 55 cm (Figure 1A). The average luminance of the stimuli was 3.1 cd/m^2^ and room ambient luminosity was between 22 and 25 Lux from the back of the screen.

The apparatus used in the experiment comprised a Tobii T120 eye-tracker (Tobii Technology, Sweden), with a sampling frequency of 120 Hz and a spatial resolution of 0.3°. The T120 model has infrared light sources and cameras embedded in the lower part of a 17-inch screen (resolution: 1,280 × 1,024 pixels), and uses corneal reflection techniques that allow the freedom of head movements. A fixation was defined as the eyes remaining in the same 30-pixel area for at least 100 ms (Manor and Gordon, 2003). Two areas of interest (AOIs) were manually defined by means of Tobii Studio software for each stimulus: the “*upper-face”* AOI (i.e., a box covering the area from the top of the forehead to the middle of the nose) and the “*lower-face”* AOI (i.e., a box covering the area from the middle of the nose to the bottom of the chin). The screen area outside these two AOIs will be referred to as “*outside-face.”* As mentioned in the Introduction, each facial emotion activates different upper-face or lower-face AUs. Specifically, “joy” and “disgust” imply more activity in the lower-face area; “anger” and “fear” mostly activate upper-face muscles; while “sadness” affects the appearance of both upper and lower face (see Figure 1B).

### 2.3. Procedure

Participants were tested individually, and after providing informed consent, they completed demographic, cognitive, affective, and visual acuity measures. Then, they were seated at approximately 55 cm from the eye-tracker's computer screen and the experimental session began with a 9-point eye-tracking calibration grid. Participants were asked to avoid large head movements as much as possible, but there were no other physical constraints. Each trial started with a 0.5 s fixation-cross in the middle of the screen, indicating that the stimulus would appear. The target stimulus was presented for 2 s (time during which eye-tracking data were recorded), and participants were instructed to look at the face in a natural manner. After 2 s, 6 emotional labels appeared at the bottom of the computer screen, and participants were asked to click on the label that best described the emotion presented. There was no time limit, and the labels were visible until the participant responded. Then, a 0.7 s black screen appeared, indicating that the participants should rest, and the next trial began. The order of stimuli presentation was pseudo-randomized, with the restriction that the same emotion or the same actor could not appear two consecutive times. Stimulus presentation and response collection (i.e., accuracy) were controlled using E-Prime presentation software (Psychology Software Tools, Pittsburgh, PA). Sessions lasted approximately 45–60 min.

### 2.4. Analysis—emotion identification

In facial emotion perception tasks, where multiple answers with forced choice paradigms are provided, there is the possibility that the participant may choose the correct emotional label by chance, which biases the accuracy rates. To avoid such biases, corresponding to the tendency to preferentially use one category of *response* in cases of doubt for a particular *stimulus*, the unbiased hit rate (Hu) (Wagner, 1993) was preferred to the percentage of correct responses (*hits*) to compute the accuracy rate for each emotion. The unbiased hit rate can vary between 0 and 1, where a hit rate of 1 indicates not only that an emotion was always identified correctly but also that the corresponding response was always used correctly (e.g., the response “anger” was only given for “anger” stimuli). The Hu index is defined as follows:

$$\text{Hu}=\frac{N_{hits}^{2}}{N_{responses}\;\times \;N_{stimuli}}$$

We computed the unbiased hit rate and then normalized the proportions with an arcsine transformation. The data were entered into an overall analysis of variance (ANOVA), with age group (younger adults, older adults) as a between-subjects factor and emotion (“joy,” “neutral,” “fear,” “anger,” “sadness,” and “disgust”) as within-subjects factor. Effect sizes are reported as partial eta-squared ($\eta_{p}^{2}$). Planned comparisons tests were conducted to further explore the interactions between age and emotion. The alpha level was set to 0.05 (levels of significance were adjusted for multiple comparisons).

### 2.5. Analysis—gaze behavior

To characterize participants' gaze behavior, three types of analysis were performed on the data collected with the Tobii eye-tracker: average fixation analysis, dynamic analysis, and pattern analysis. Each of them is explained in detail in this section.

#### 2.5.1. Averaged fixations analysis

Firstly, to provide average statistics about gaze behavior during the target face presentation, the mean number of fixations (i.e., fixation count) and the total fixation duration (in seconds) within the AOIs were calculated using Tobii Studio software. Blinks and saccades were excluded from the analysis. The data were entered into an overall analysis of variance (ANOVA), with age group (younger adults, older adults) as between-subjects factor, and AOIs (upper-face and lower-face areas) and emotion (“joy,” “neutral,” “fear,” “anger,” “sadness,” and “disgust”) as within-subjects factor.

#### 2.5.2. Dynamic analysis

Average fixation statistics have been widely used in the literature to characterize gaze behavior. However, they fail to describe important spatio-temporal dynamics, such as the spatial distribution and time course of fixations. To further explore gaze behavior dynamics in younger vs. older adults, a deeper analysis of eye movement data was performed in a second step. Concretely, we computed the percentage of participants from a given age group looking at each AOI for each time instant *t*_*i*_ of the 2 s stimuli watching. As a result, we built *aggregated dynamic behavior time-series*, which are double timelines (one per AOI: *upper-face* and *lower-face*) using a graded color-scale to codify the percentage of participants looking at each AOI across time. The time-series were obtained by aggregating the information per type of emotional stimuli and age group.

#### 2.5.3. Pattern analysis

**Figure 2:**
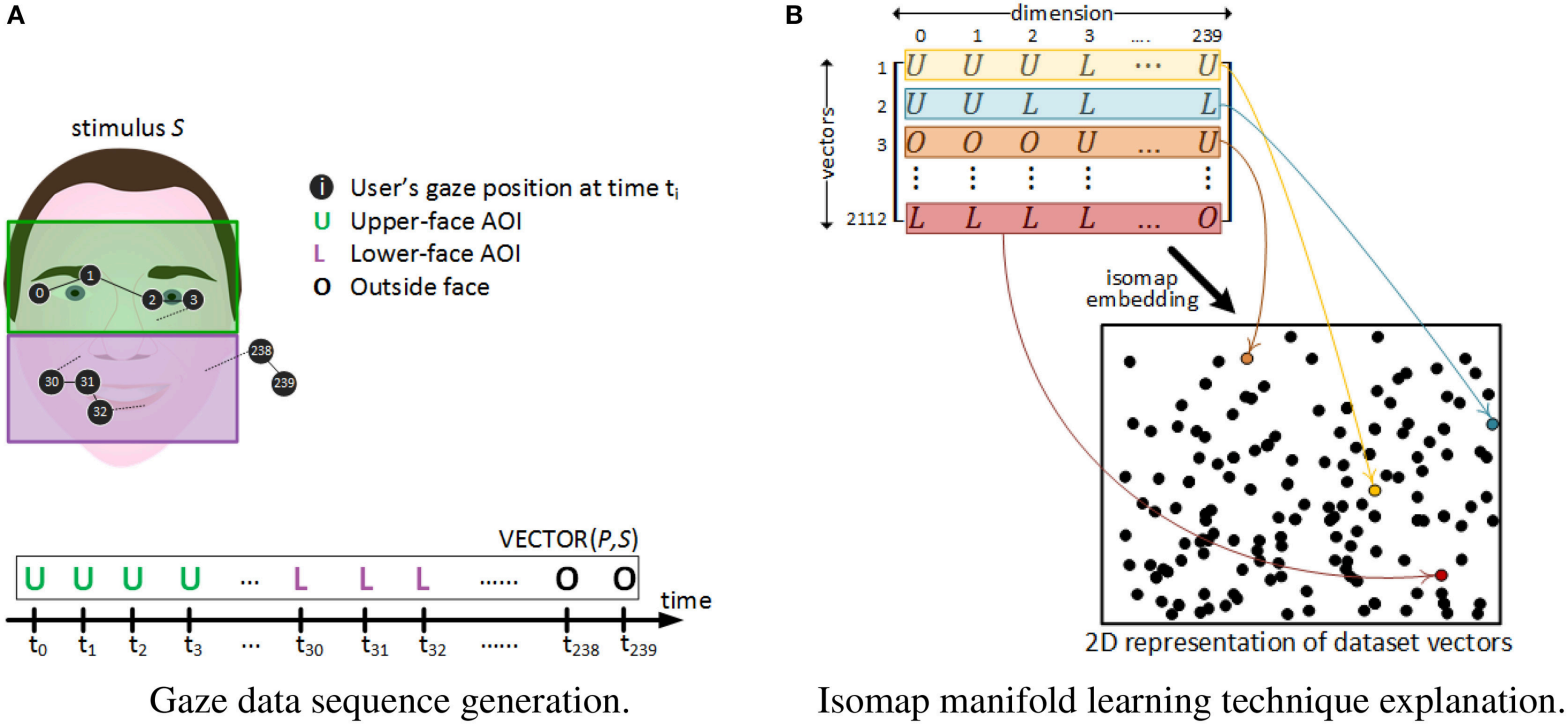
**Gaze dataset vectors' generation and their 2D isomap projection. (A)** A gaze behavior sequence for a given participant *P* watching stimulus *S* for 2 s, is codified as a vector of 240 letters. **(B)** Gaze vectors' dimension is reduced from 240 to 2. Each black dot in the 2D space corresponds to the 2D location of each of the 2,112 vectors after dimensionality reduction. As an example, 4 vectors are colored in yellow, blue, brown and maroon respectively, and their assigned 2D point is colored accordingly in the 2D space.

Dynamic analysis is a per-group averaged representation devoted to reveal common AOIs exploration trends over time. Conversely, pattern analysis aims at grasping individual gaze behavior differences and similarities over time between participants. Patterns are defined here as sequences of spatial switches between AOIs that are repeated across trials. Following the approach by Cristino et al. (Cristino et al., 2010; Hansen et al., 2015), for each time instant *t*_*i*_ of stimuli watching, the participant's eye fixation was labeled with a letter (“U” for *upper-face*, “L” for *lower-face* and “O” for *outside-face*). More precisely, as participants looked at each stimulus for 2 s with eye-tracking information being extracted at a sampling rate of 120 Hz, each trial was represented as a time-ordered vector of 240 letters (see Figure 2A). Thus, a total of 44(*stimuli*) × 48(*participants*) = 2, 112 vectors were created from our experimental data. Differently from Cristino et al., that propose the separate analysis of each pair of vectors to obtain metrics of behavioral similarity between two trials, we extracted patterns from the whole 2112 vectors collection. This was achieved by applying dimensionality reduction methods and clustering techniques as follows.

The visual inspection of such a large amount of high-dimensional information (i.e., 2,112 vectors of 240 dimensions) is noisy and its direct interpretation would have been difficult. One way to overcome this problem is to reduce the dimensionality of data using *manifold learning* methods (Vlachos et al., 2002). Here, we used the *Isomap* algorithm provided by the *Scikit-Learn* toolkit (Pedregosa et al., 2011). It has the advantage of seeking for a lower-dimensional embedding, typically in a 2D space for visualization purposes, which preserves the original intrinsic geometry of the data (Tenenbaum et al., 2000). Thus, each of the 2,112 vectors (i.e., each trial) was represented as a point in a 2D space, as depicted in Figure 2B. Consequently, points representing trials with similar spatio-temporal gaze patterns should tend to be closer in this 2D space, while trials with very different patterns should be much more distant.

Finally, to capture inter- and intra-group differences and similarities in spatio-temporal gaze patterns, we clustered the obtained 2D points using the K-means clustering algorithm (Hartigan and Wong, 1979; Pedregosa et al., 2011). K-means is a commonly used partitioning method for splitting a points cloud into a set of *K* groups (called “clusters”). It is an unsupervised method, as it does not take into account a priori knowledge about the data (in that case, whether a given point belongs to an older or a younger adult trial) to group the points. The analysis consists in (i) setting K to 2, and then (ii) analyzing the distribution of younger and older adults' patterns within these two clusters. The objective pursued using this technique is to provide quantitative metrics about how grouped or dispersed are the different individuals' behaviors. For example, if one of the found clusters mostly contains younger adults' points while the other mainly groups older adults' ones, it would mean that each age group's behavior is separable, leading to high inter-group and low intra-group gaze behavior differences; however, if the two found clusters contain a high percentage of both age groups points, it would mean that inter-group behavior is not so separable and imply the existence of intra-group differences.

Overall, our approach consisting in computing sequences of gaze behaviors, dimension reduction and clustering allows to extract inter- and intra-group differences and similarities of spatio-temporal gaze patterns.

## 3. Results

Analysis of participants' demographic characteristics (see Table 1) revealed that the younger and older adults groups did not differ significantly (all *p* > 0.05) on the measures of years of education, depression (BDI-II) or facial perception skills (BFRT).

To control for potential gender differences in emotion identification (Hall et al., 2000), this variable was initially entered as a between-subject factor in the analyses. However, as gender failed to yield any significant main effects (*F* < 1) or interactions (*p* > 0.2), so we collapsed across gender in the reported analysis. In addition, to also examine whether there were any differences for male vs. female faces, or for young vs. old faces (for example, see Ebner et al., 2011), these variables were also initially entered as between-subject factors in the analyses, but as no significant effects were found across all measures (all *p* > 0.05) and we collapsed for sex/age of the faces in the following analysis.

### 3.1. Emotion identification data

Overall, both younger and older adults obtain high scores on the emotion identification task. The mean percentage of correct responses of younger adults was 96.50% and that of older adults reached 90.53%. While the few confusions by younger adults were evenly distributed among positive and negative emotions, older adults clearly tended to mix-up negative emotions. For instance, the emotional categories most often confused by older adults were “fear” (confused with “anger” in 9.66% of cases) and “anger” (confused with “disgust” in 5.11% and with “sadness” in 4.55% of cases). The “neutral” emotion was never confused with “joy,” but was confused with “sadness” (3.41% of the cases) and “anger” (2.84% of cases).

**Figure 3:**
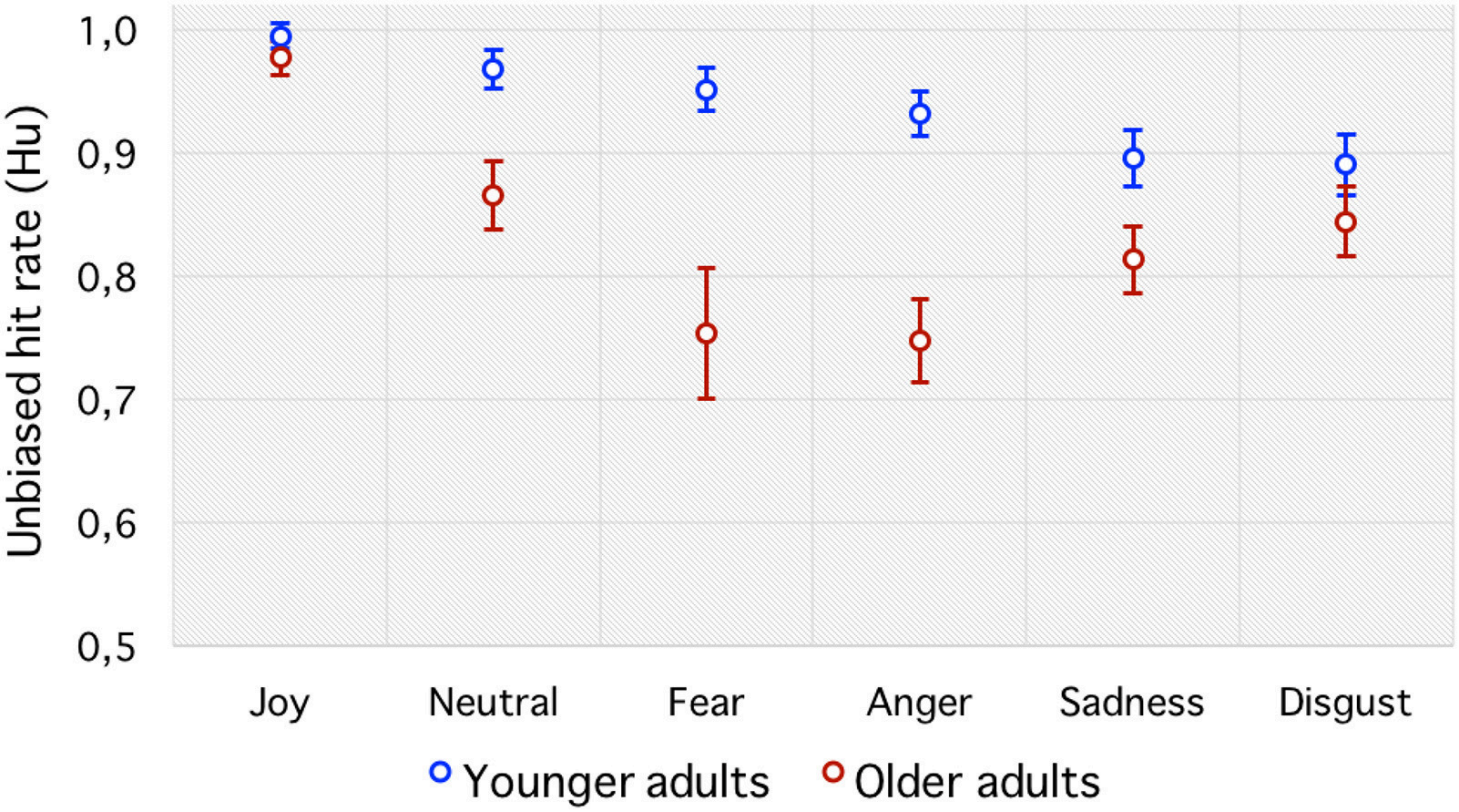
**Unbiased hit rate (Hu)**. Emotion recognition accuracy for each emotion category in younger and older adults. Error bars indicate standard errors of the means.

To avoid such biases, the unbiased hit rate (Hu) was preferred. It revealed a significant main effect of age group [$F_{\left(1,\;42\right)}=17.59,\;p<0.001,\eta_{p}^{2}=0.30$], with young adults (0.93 ± 0.02) showing greater emotion perception accuracy than older adults (0.83 ± 0.03). There was also a significant main effect of emotion, *F*_(5, 210)_ = 22.21, *p* < 0.001, $\eta_{p}^{2}=$ 0.34, which was qualified by the predicted age group by emotion interaction, *F*_(5, 210)_ = 5.80, *p* < 0.001, $\eta_{p}^{2}=$ 0.12 (see Figure 3). Follow-up planned comparisons revealed significant age differences, with older adults performing more poorly than younger adults at identifying “fear” (*p* < 0.001) and “anger” (*p* < 0.001) with a tendency for “neutral” (*p* = 0.01) and “sadness” (*p* = 0.03), but not “joy” (*p* = 0.26) or “disgust” (*p* = 0.25).

### 3.2. Gaze behavior data

Gaze behavior related results are reported at three levels, each corresponding to one of the three types of analysis presented in Section 2.5: (1) average fixation level, which includes mean number and total duration of fixations; (2) dynamic level, that provides aggregated dynamic behavior time series; and (3) pattern level, that represents and clusters individual gaze trials in a 2D space.

#### 3.2.1. Averaged fixations level

**Figure 4:**
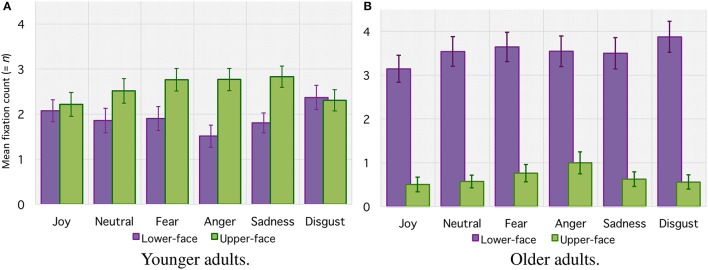
**Mean number of fixations (=*n*) within lower-face and upper-face AOIs for each facial emotion category for younger adults (A)** and older adults **(B)**. Error bars indicate standard errors of the means.

For the *mean number of fixations* (see Figure 4), the ANOVA revealed no significant main effect of age group (with 4.2 ± 0.06 fixations for older adults vs. 4.5 ± 0.05 for younger adults, *F*_(1, 42)_ < 1. There was a significant main effect of emotion [*F*_(5, 210)_ = 5.19, *p* < 0.001, $\eta_{p}^{2}=$ 0.11] and AOI [*F*_(1, 42)_ = 15.47, *p* < 0.001, $\eta_{p}^{2}=$ 0.27], together with an interaction between age group and AOI [*F*_(1, 42)_ = 37.42, *p* < 0.001, $\eta_{p}^{2}$ = 0.27]. Planned comparison revealed that older adults made more fixations over the *lower-face area* than over the *upper-face area* (respectively 3.5 ± 0.03 vs. 0.7 ± 0.02, [*F*_(1, 42)_ = 51.53, *p* < 0.001]. For younger adults the difference between the number of fixations over the *lower-face area* and *upper-face area* was marginal and did not reach significance [respectively 1.9 ± 0.03 vs. 2.6 ± 0.02, *F*_(1, 42)_ < 1]. Finally, there was an interaction between age group, emotion, and AOIs [*F*_(5, 210)_ = 4.61, *p* = 0.01, $\eta_{p}^{2}$ = 0.09]. This result indicates that older adults made more fixations over the *lower-face* area for each emotion (all *p* < 0.001), whereas for for younger adults the number of fixations over each area depended on the emotional category. Younger adults made more fixations in the *upper-face area* for “anger” (*p* = 0.007) and “sadness” (*p* = 0.013), with a tendency for “fear” (*p* = 0.06), whereas differences for “joy,” “neutral” nor “disgust” did not reach significance (all *p* > 0.1).

**Figure 5:**
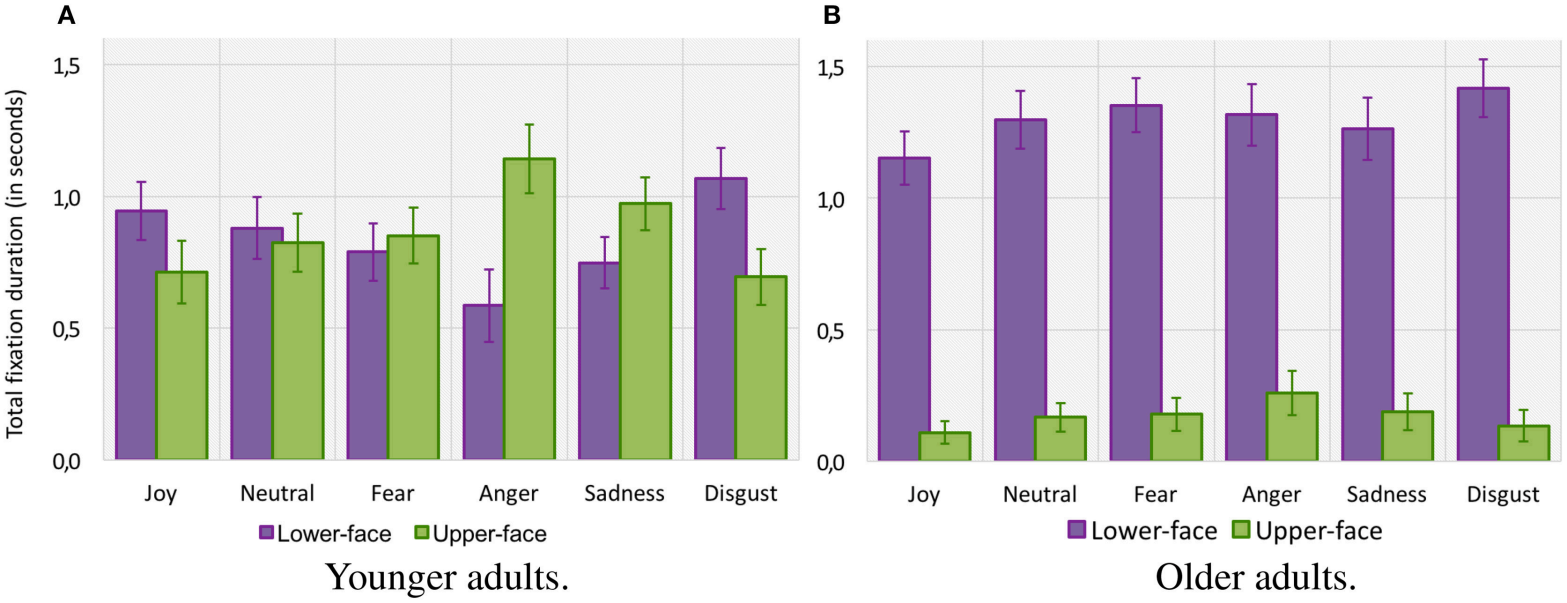
**Total fixation duration *(in seconds)* within lower-face and upper-face AOIs for each facial emotion category for younger adults (A)** and older adults **(B)**. Error bars indicate standard errors of the means.

For the *total fixation duration* (see Figure 5), the ANOVA indicated a main effect of age group [*F*_(1, 42)_ = 5.85, *p* = 0.01, $\eta_{p}^{2}=$ 0.12], suggesting that older adults tended to fixate the target emotional faces for a shorter duration (1.5 ± 0.01 s) than younger adults (1.7 ± 0.01 s). The analysis also indicated a main effect of emotion [*F*_(5, 210)_ = 7.19, *p* < 0.001, $\eta_{p}^{2}=$ 0.15] and AOI [*F*_(1, 42)_ = 20.76, *p* < 0.001, $\eta_{p}^{2}=$ 0.33], together with an interaction between age group and AOI [*F*_(1, 42)_ = 22.97, *p* < 0.001, $\eta_{p}^{2}$ = 0.35]. However, planned comparison revealed that whereas older adults fixated the *upper-face area* for a shorter time than younger adults (respectively 0.2 ± 0.01 s vs. 0.9 ± 0.01 s, *F*_(1, 42)_ = 36.75, *p* < 0.001), they fixated the *lower-face area* longer than younger adults [respectively 1.3 ± 0.01 s vs. 0.8 ± 0.01 s, *F*_(1, 42)_ = 11.48, *p* < 0.001]. Planned comparisons also indicated that the difference between the total fixation duration over the *lower-face area* and *upper-face area* was significant in older adults (*p* < 0.001) but not in younger adults (*F* < 1). Finally, there was an interaction between age group, emotion, and AOI [*F*_(5, 210)_ = 4.69, *p* < 0.001, $\eta_{p}^{2}=$ 0.10], showing that older adults fixated the *lower-face* area longer for each emotion (all *p* < 0.001), whereas for younger adults fixation duration over each area depended on the emotional category. Younger adults fixated the *upper-face area* a longer time for “anger” (*p* < 0.001), with a tendency for “sadness” (*p* = 0.07), but they fixated the *lower-face area* a significantly longer time for “disgust” (*p* = 0.02). Differences between the *upper* vs. *lower-face area* did not reach significance for “fear,” “joy,” or “neutral” (all *p* > 0.05).

#### 3.2.2. Dynamic level

**Figure 6:**
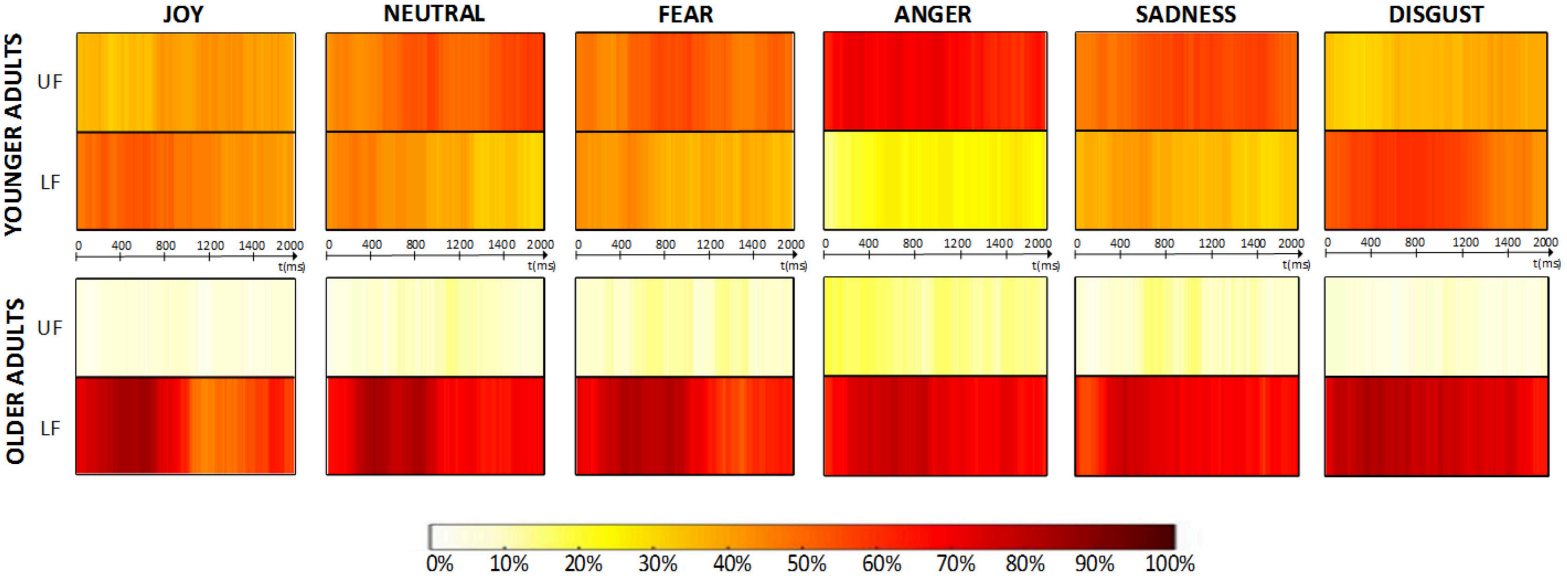
**Dynamic analysis results**. *Aggregated dynamic behavior time-series* obtained for each type of emotional stimuli are presented. The percentage of participants looking at each AOI (UF = “upper-face”; LF = “lower-face”) at each time *t*_*i*_ is coded using a graded color-scale.

Concerning gaze spatio-temporal dynamics, Figure 6 depicts the *aggregated dynamic behavior time-series* obtained from collected the data. The time-series show that the distribution of average fixation over *upper-face* and *lower-face* AOIs found in Section 3.2.1 was evenly maintained across time, both for younger and older adults. We define three *consensus* levels (*low, medium* and *high*), depending on the percentage of participants looking at the same AOI at a given time *t*_*i*_ (respectively: <50%, in the range 50–70% and >70%). Older adults showed a global *medium-to-high consensus* while exploring the *lower-face area* throughout the 2 s timeline and for each emotion, whereas younger adults never exceeded a *medium consensus* level for any AOI and emotion, except for “anger” which was the only emotion presenting a *high consensus* over time on the *upper-face* area.

#### 3.2.3. Pattern level

**Figure 7:**
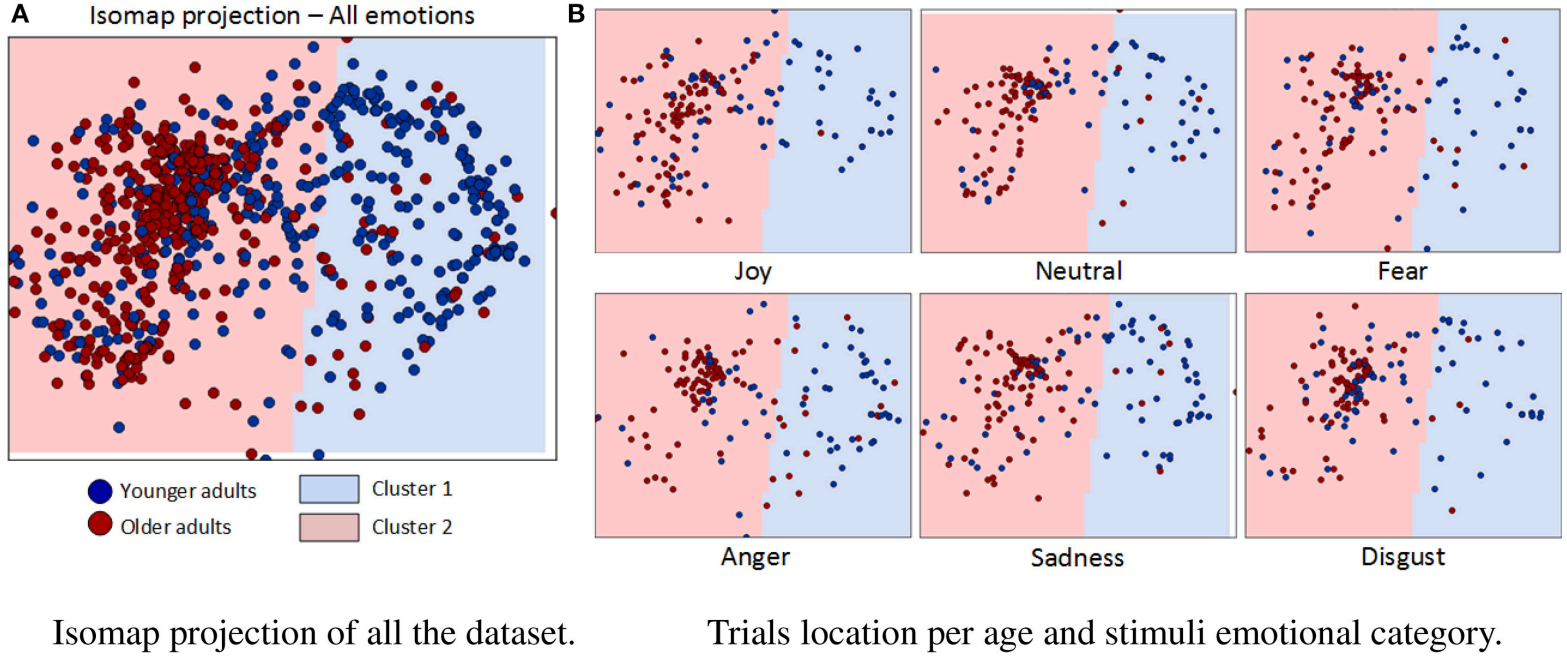
**Isomap projection and clustering results. (A)** Each point in the 2D space corresponds to one of the 2112 trials and is colored in dark-blue or dark-red, depending on whether it was performed by a younger or older adult. Each obtained cluster (“cluster 1” and “cluster 2”) is presented as background light-blue and light-red colors, respectively. **(B)** Trials belonging to each stimuli emotional category are shown in a separate figure.

Figure 7 shows the result of Isomap projection and clustering of dataset gaze vectors, globally (a) and per basic emotion (b). Each point in the 2D space corresponds to one of the 2,112 trials and is colored in dark blue or dark red, depending on whether it was performed by a younger or older adult, respectively. The boundaries of the two clusters obtained are shaded in light blue (“cluster 1”) and light red (“cluster 2”). Table 2 summarizes the percentage of trial points that belong to each cluster, depending on the age of participants and the type of emotional stimuli shown. Clustering reveals that 92% of older adults' instances belong to the same cluster (“cluster 2”), while younger adults' trials are equally divided between the two clusters (48% in “cluster 1” and 52% in “cluster 2”). Interestingly, when results are analyzed per type of emotional stimulus, a significantly higher overlap between clusters is found for 2 emotions: “joy” (64% of younger adult and 98% of older adult instances lie in “cluster 2”) and “disgust” (63% of younger adult and 94% of older adults trials lie in “cluster 2”). Conversely, the lowest overlaps were found for “anger” (62% of younger adults' trials in “cluster 1”; 85% of older adults' trials in “cluster 2”), “sadness” (55% of younger adults trials in “cluster 1”; 93% of older adults' trials in “cluster 2”) and “fear” (51% of younger adults' trials in “cluster 1”; 92% of older adults' trials in “cluster 2”). For “anger” it is also important to mention that there is an increase in the percentage of older adult instances belonging to “cluster 1” (15%) with regard to other emotions.

**Table 2:** **Clustering results**.

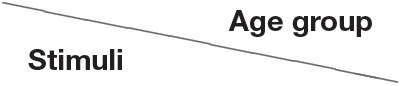	**Cluster 1**		**Cluster 2**	
	**YA (%)**	**OA (%)**	**YA (%)**	**OA (%)**
Joy	36	2	64	98
Neutral	48	10	52	90
Fear	51	8	49	92
Anger	62	15	38	85
Sadness	55	7	45	93
Disgust	37	6	63	94
All emotions	48	8	52	92

*The percentage of trials belonging to each cluster is indicated, per age of participants (YA, Younger Adults; OA, Older Adults) and type of emotional stimuli shown*.

## 4. Discussion

The ability to accurately identify others emotions is critical for everyday communication, and in turn well-being and mental health. In the recent years there has been considerable concern that this capacity may decline with age (Ruffman et al., 2008; Chaby et al., 2015; Pierguidi and Righi, 2016). However, in order to interpret age-related changes in facial emotion identification, most research has typically focused on socio-emotional or aging-brain model explanations, but more perceptual differences in the gaze strategies that accompanied facial emotional processing with advancing age have been under-explored. The present study used eye-tracking technology, which provides direct information about how and when a visual stimulus is perceived, while younger and older adults were engaged in a task-oriented situation of facial expression identification. The first research question of the present study aimed to increase knowledge on how gaze behavior could explain differences and similarities underlying facial expression identification in the two groups. The second research question investigated inter- and intra-group differences and similarities in gaze exploration strategies. Thus, in addition to performance measures and conventional analysis of gaze fixations, we examined spatio-temporal patterns of gaze behavior. Our results provide evidence that converges with previous observations and also report several novel findings about facial emotion processing and gaze strategies with advancing age, that will be discussed in more detail below.

### 4.1. Age differences in facial emotion identification

After controlling for response biases, it appeared that the identification of the facial expressions of “joy” and “disgust” is preserved with age. On the contrary, “fear” and “anger” resulted especially difficult to identify for older adults, while they found moderated difficulties at detecting “sadness” and “neutral” facial emotions. Similar preservation and difficulties with aging, predominantly using forced-choice tasks and acted static facial pictures, have been previously reported in the literature though not consistently. For example, some previous research found deficits in recognizing “sadness,” but not “fear” nor “anger” (e.g., Suzuki et al., 2007), whereas other studies found older adults' difficulties in recognizing “fear” and “anger,” but not “sadness” (e.g., Calder et al., 2003; Circelli et al., 2013). Finally, some studies found impairments for all the three emotions of “fear,” “anger,” and “sadness” (Wong et al., 2005; Grainger et al., 2015). The fact that older adults showed better expression identification for “joy” and “disgust” is consistent with other reports (Ruffman et al., 2008). It should be noted that “joy” was the only positive emotion, so participants were able to make the correct choice as soon as they recognized a smile, and its identification was near ceiling in both younger and older adults. Thus, using only a few basic emotional categories allowed participants to choose their response based essentially on exclusion rules, which is less likely to be the case in real life (Frank and Stennett, 2001). In this study we focused on basic emotions, but a possible way to avoid this limitation would have been to use an alternative facial expression database (e.g., the Geneva Multimodal Emotion Portrayal—GEMEP; Bänziger and Scherer, 2010) with a more balanced number of positive and negative emotions. In any case, in this study participants knew they would be confronted with a forced-choice emotion identification task after a 2-s exploration of each facial picture, and consequently adopted a gaze behavior strategy to handle the problem. The main focus of this work was indeed the analysis of such adopted gaze exploration strategies, more than evaluating their ability to correctly decode facial expressions.

### 4.2. How older and younger adults explore emotional faces

Traditional analyses of gaze fixations (i.e., number and total duration of fixations) and where on the face these fixations occurred (i.e., *lower-face* or *upper-face area*) revealed some differences between age groups. While older and younger adults overall made the same number of fixations on faces, they differed significantly in where these fixations were made. Older adults made consistently more fixations and for a longer duration over the *lower-face area* when exploring all emotions, whereas in younger adults, gaze behavior was influenced by the type of emotional stimulus. They made more fixations over the *upper-face area* for “anger,” “sadness,” and “fear” faces, whereas fixations were more equally distributed between the two AOIs for “joy,” “disgust,” and “neutral” faces. However, younger adults fixated longer the *upper-face area* for “anger” and “sadness” and the *lower-face area* for “disgust,” whereas the duration of fixation was distributed over these two areas for “joy,” “fear,” and “neutral.” This age-related tendency to fixate more frequently and for a longer time the *lower-face area* is consistent with previous findings (Wong et al., 2005; Sullivan et al., 2007) which demonstrated under-attention for eyes and over-attention for the mouth in older adults when viewing facial expressions. In the future, however, it would be relevant to go beyond measures of gaze fixations over AOIs by using the recent iMap approach (Caldara and Miellet, 2011) that extracts fixations from raw data on their exact spatial location and then statistically compares between conditions or groups with a much finer spatial resolution.

### 4.3. Spatio-temporal dynamics of gaze behavior during aging

The aforementioned results provide information about each group's overall gaze behavior, but not about underlying spatio-temporal dynamics. For example, younger adults could have been able to rapidly identify at the very beginning of the stimulus presentation the more relevant area of the face to focus, whereas this process could have taken more time for older adults. It may also have been possible that participants initially took a time to explore both lower and upper parts of the face to, then, chose a given area to focus. However, our spatio-temporal dynamic analysis indeed revealed that the focus toward the *lower-face area* for older adults, and the more distributed gaze into the *upper-face* and *lower-face* areas in younger adults, were evenly maintained over time.

These traditional and dynamic analyses of gaze behavior shed light on the reasons behind older adults' performances in the facial emotional identification task. According to the Facial Action Coding System (FACS, Hager et al., 2002) the facial expressions of “joy” and “disgust” activates specific AUs that mostly involve lower-face muscles; “fear” and “anger” activates AUs that involve upper-face muscles, whereas “sadness” activates at similar levels upper and lower face muscles. Interestingly, computational methods for automatic facial expression recognition assigned weights to the most relevant facial areas essential to correctly detect each facial emotion (e.g., Lucey et al., 2010; Maalej et al., 2011). Results are in line with the FACS: the highest weights for “joy” and “disgust” belong to the mouth region, those for “fear” and “anger” appear in the upper-face, while “sadness” expression has high weighting both around the eyes and the nose. Moreover, it has been recently hypothesized (Srinivasan et al., 2016) that to visually interpret facial expressions, our brain must identify which AUs are activated in a face and this could be mediated by the *posterior Superior Temporal Sulcus* (pSTS) which plays a crucial role in the analysis of changeable aspects of the face (Haxby et al., 2000). In other words, this distribution of specific AUs associated with the tendency of older adults to fixate the lower-face region over time, may explain their identification performances. While “joy” and “disgust” were accurately detected, “fear” and “anger” identification could not be as accurate as crucial facial areas were not/scarcely explored. The case of “sadness” is particularly interesting as it is the only negative emotion activating both upper and lower facial muscles at the same intensity, which explains the better identification rate.

Our dynamic analyses also revealed that older adults reached high consensus levels looking at the *lower-face* all throughout the trial. Together with the low number of fixations found on the *upper-face*, this suggests that they seldom switched to the *upper-face*. However, no strong conclusions can be drawn on this basis for younger adults, as several fixations occurred over each face area and high consensus levels were not reached inside any period within the 2 s timeline. Therefore, these analyses did not reveal how fixations were spatially distributed over time nor common gaze exploration trends in given time periods, suggesting important intra-group gaze behavior differences for younger adults. The only exception arose for the emotion “anger,” which presented a high consensus level throughout the 2 s and a significantly higher number of fixations in the *upper-face*, indicating a consistent gaze behavior for this emotion in younger adults.

### 4.4. Individual differences and age-group strategies in gaze behavior

In order to better target individual differences and similarities in gaze behavior during facial expression processing, gaze patterns for each trial were represented in a 2D space and then separated into two clusters. Overall, we found that trials corresponding to older adults were clearly clustered together whereas trials corresponding to younger adults were dispersed in the 2D space. The highest overlap was found for “joy” and “disgust” emotions, for which the majority of younger adults' trials lied in the same cluster as older adults' ones, meaning that for these emotions younger adults' gaze behavior tended to be closer to that of older. However, “fear,” “anger,” and “sadness” presented the lowest overlap levels, which means that the behavior of younger and older adults was significantly different for these emotions. It is interesting to notice that older did not shift toward younger adults' gaze behavior for any emotion (excepting, very slightly, for “anger”). This demonstrates that gaze behavior patterns of older adults are more consistent than those of younger adults. This may suggest that older adults present a *focused-gaze* strategy, consisting in focusing only on the lower part of the face during the 2-s display. By contrast, younger adults appear to be more variable: they may use very different strategies, overall and also in adaptation to each emotion, such as *focused-gaze* strategy that is more extensively used by older adults or a more *exploratory-gaze* strategy consisting in constantly exploring the upper and lower parts of the faces and switching between these two parts during the 2-s display.

Possible explanations for the fact that older adults' gaze behavior is more consistent (less variable) may come from other social and physical changes commonly developed with aging. For instance, it is well-known that eye-to-eye contact is less common with social partners for older populations in daily life (Slessor et al., 2010, 2016). Another possibility is that older adults are mostly attracted to the mouth because it offers relevant social signals such as voice production and lip-reading during social communication and interaction, that could constitute, with advancing age, a compensatory strategy to maximize the amount of information available due to a discrete hearing loss (Cienkowski and Carney, 2002). Thus, even though in the present study facial stimuli were static and lips were not moving, this acquired compensatory strategy would have made the mouth a salient facial feature for older adults. An alternative explanation could arise from changes in posture with age. Hyper-kyphosis, that commonly affects posture with advancing aging (Kado, 2009) has been associated with a forward head posture in sitting position (Kuo et al., 2009) that may lead to a gaze orientation toward the lower-area of the faces in the computer screen.

One interesting final explanation for our results could emerge from age-related changes in the use of “bottom-up” and “top-down” visual attention strategies. Facial emotional stimuli processing is mediated by both “bottom-up” (sensory driven mechanisms that select stimuli based on their physical salience) and “top-down” factors (which select stimuli based on expectations, knowledge and goals) that operate interactively (Buschman and Miller, 2007; Sussman et al., 2016). It has been shown that the interplay between “bottom-up” and “top-down' processes changes in favor of the latter with age (Açık et al., 2010). The data reported here are consistent with the idea that “bottom-up” influences of salient facial features (such as wrinkles and facial appearance changes caused by the activation of AUs) may lose strength with age. While younger people could unconsciously and randomly be attracted by “bottom-up” processes to the most pertinent areas of the face (i.e., those where appearance changes show-up), it would not be the case for older adults. Consequently, these differences in visual attention processing patterns could have (i) strongly impacted emotion identification performances in favor of younger adults and (ii) fostered a more consistent gaze behavior in older adults.

## 5. Conclusion

To conclude, the presented work went beyond traditional eye-tracking statistics by studying gaze behavior dynamics and patterns. This allowed to emphasize that, confronted to emotional faces, younger and older adults do not prioritize or ignore the same facial areas. Older adults mainly adopted a *focused-gaze* strategy, consisting in focusing only on the lower part of the face during the 2 s stimuli display. This consistency may constitute a robust and distinctive “social signature” of emotional identification in aging. Younger adults, however, were more dispersed in terms of gaze behavior and used a more *exploratory-gaze* strategy, consisting in repeatedly visiting both facial areas.

## Author contributions

Study concept and design was performed by LC and MC. LC and VL run the experiment. LC, IH, and MA did the implementation of data analysis and obtained the results. LC and IH did the interpretation of data. LC, IH, VL, and MC wrote the paper.

## Funding

This work was partially supported by the Labex SMART (ANR-11-LABX-65) under French state funds managed by the ANR within the Investissements d'Avenir program under reference ANR-11-IDEX-0004-02. This work also received support from ROMEO2 project.

### Conflict of interest statement

The authors declare that the research was conducted in the absence of any commercial or financial relationships that could be construed as a potential conflict of interest.

## Footnotes

^1^It requires participants to match a series of unfamiliar target faces from a six-stimuli array in different orientations and lighting conditions.

^2^This database does not include faces expressing “surprise”; indeed, for still pictures and without any contextual information, the facial expressions of “surprise” and “fear” are frequently confused, even by younger adults (Isaacowitz et al., 2007).
